# Atomistic Simulations Reveal Crucial Role of Metal Ions for Ligand Binding in Guanidine‐I Riboswitch

**DOI:** 10.1002/marc.202400606

**Published:** 2024-09-03

**Authors:** Leon Franke, Christoph Globisch, Mehmet Can Karakurt, Theresa Stephan, Christine Peter

**Affiliations:** ^1^ Department of Chemistry University of Konstanz Universitätsstraße 10 78457 Konstanz Germany

**Keywords:** guanidinium, ion triad, molecular dynamics, riboswitches, RNA structure

## Abstract

Riboswitches are structured ribonucleic acid (RNA) segments that act as specific sensors for small molecules in bacterial metabolism. Due to the flexible nature of these highly charged macromolecules, molecular dynamics simulations are instrumental to investigating the mechanistic details of their regulatory function. In the present study, the guanidine‐I riboswitch serves as an example of how atomistic simulations can shed light on the effect of ions on the structure and dynamics of RNA and on ligand binding. Relying on two orthologous crystal structures from different bacterial species, it is demonstrated how the ion setup crucially determines whether the simulation yields meaningful insights into the conformational stability of the RNA, functionally relevant residues and RNA‐ligand interactions. The ion setup in this context includes diffuse ions in solution and bound ions associated directly with the RNA, in particular a triad of 2 Mg^2+^ ions and a K^+^ ion in close proximity to the guanidinium binding site. A detailed investigation of the binding pocket reveals that the K^+^ from the ion triad plays a decisive role in stabilizing the ligand binding by stabilizing important localized interactions, which in turn contribute to the overall shape of the folded state of the RNA.

## Graphical abstract

Riboswitches regulate gene expression in bacteria upon selective binding of small molecules. In molecular dynamics simulations, the guaninidium‐I riboswitch from *D.dadantii* consistently loses its ligand. By placing a metal ion triad from an orthologous riboswitch at the binding pocket, the binding is stabilized. The key player is K^+^ from the triad, which stabilizes localized interactions.

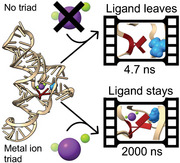

## 1 Introduction

Riboswitches are structured segments of mRNA used by bacteria and archaea to regulate gene expression. They act as sensors for small molecules, such as metabolites and ions, and regulate the expression of down‐stream gene products in *cis*.^[^ ^1^ ^]^ One class of such riboswitches is the *ykkC* riboswitch family. The members of this family (guanidine‐I, II, III, and IV) have been shown to be specific sensors for increased levels of intracellular guanidine (Gd).^[^ ^2^, ^3^, ^4^, ^5^ ^]^ Under intracellular conditions, the strongly basic Gd forms the guanidinium cation (Gd^+^). Upon interaction with Gd^+^, the Gd riboswitches (Gd RS) turn on genes involved in Gd export and breakdown, suggesting that regulating intracellular Gd levels is an important piece of bacterial metabolism.^[^ ^2^ ^]^ After the ligand for the formerly orphaned class of RS was identified, structural biologists were exceptionally fast to solve the structure of three members of the Gd RS family (Gd‐I, II, and III).^[^ ^6^, ^7^, ^8^, ^9^, ^10^, ^11^ ^]^ These studies revealed that the three Gd RS have distinct tertiary folds, yet with remarkably similar Gd^+^ binding pockets, hinting toward a process of independent but convergent evolution.^[^ ^12^ ^]^ While the crystal structures provide insights into the structural underpinnings of the sensing of Gd^+^, mechanistic details of the interplay between ligand binding and RNA conformational changes and the resulting regulatory function of these RS remain poorly understood. Here, molecular dynamics (MD) simulations are an indispensable tool that complements structural methods.^[^ ^13^, ^14^, ^15^ ^]^ MD simulations have been successfully used to investigate ligand binding and RNA conformational dynamics for several RS systems.^[^ ^16^, ^17^, ^18^, ^19^, ^20^, ^21^ ^]^ Recently, a combination of atomistic MD simulations and machine learning analysis methods have been used to identify ligand dependent conformational changes of the Gd‐II RS and propose a mechanism that couples ligand binding to switching.^[^ ^22^ ^]^

**Figure 1:**
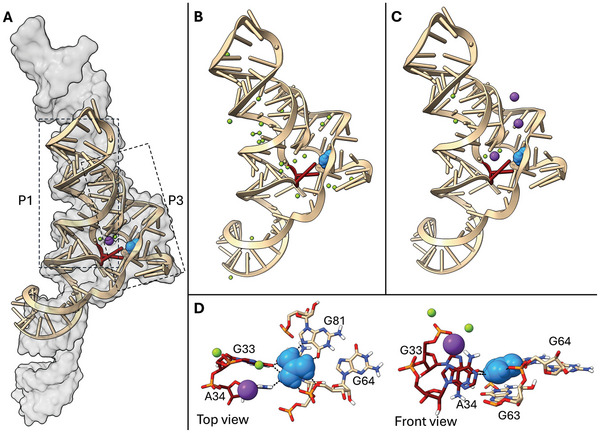
A) Crystal structures of Gd‐I riboswitch orthologs: Structure of Gd‐I from *D.dadantii* (5U3G) is shown as tan cartoon, Gd‐I from *Burkholderia* (7MLW) is shown as gray surface. Dashed boxes indicate the RNA loops P1 and P3. Residues A34 and G33 (the AG‐bulge, red) flip out of P1 to bind into P3. The Gd^+^ ligand (blue) from 5U3G, and the metal triad (K^+^ (violet), 2 Mg^2+^ (green) from 7MLW are shown for orientation. B) Simulation starting structure for 5U3G with original ion setup: Gd^+^ (blue) and Mg^2+^ ions (green) were adopted from crystal structure. C) Simulation starting structure for 5U3G with altered ionic setup: K^+^ ions (violet) and Mg^2+^ ions (green) were transferred from 7MLW, Gd^+^ was left unchanged. D) Detailed top view (left) and front view (right) of the binding pocket with all residues taking part in ligand binding and the transferred ion triad. Ligand binding interactions are shown as dashed black lines, other coloring as above.

Following our approach for the Gd‐II RS, we decided to study the molecular dynamics of the Gd‐I RS, which comprises 75% of all known *ykkC* examples.^[^ ^8^ ^]^ Thus, we set up atomistic MD simulations of the Gd‐I RS from the bacterium *Dickeya dadantii* based on the crystal structure solved by Battaglia et al. in 2017^[^ ^6^ ^]^ (from here on referred to by its PDB code 5U3G). We expected the Gd^+^ ligand to be similarly tightly bound to its pocket as in the Gd‐II RS. Strikingly, in the majority of our simulations, the ligand did not remain in the binding pocket, despite applying careful equilibration protocols. This was surprising given the reported Gd^+^ affinity of 39 µm.^[^ ^6^ ^]^ While searching for an explanation for this somewhat unsettling observation, we looked at the crystal structures of two orthologous Gd‐I RS from different bacterial species, namely *Sulfobacillus acidophilus* (PDB Code 5T83^[^ ^7^ ^]^), and *Burkholderia sp. TJI49* (PDB code 7MLW^[^ ^8^ ^]^). In particular, the 7MLW structure by Trachman et al.^[^ ^8^ ^]^ was interesting in our context since the authors reported an essential difference to the other two: The specific placement of a K^+^ ion in a position that forms an uncommon metal triad in close proximity to the binding pocket. While the two Mg^2+^ ions of the triad are present and in similar positions in the other orthologs, this K^+^ is absent. A comparison of the Gd‐I RS investigated here (5U3G) and the RS in which the triad was found (7MLW) is shown in **Figure** **1**A. While 7MLW (shown in gray) is notably larger than 5U3G (shown in tan), the binding pockets of both RS not only share the same global architecture but they also share the same sequence of residues. Since the pocket residues convey specificity and affinity for Gd^+^, their conformational arrangements are fairly similar as well. Trachman et al. state that the triad with the additional K^+^ ion plays a critical role in stabilizing the ligand binding in their riboswitch ortholog.^[^ ^8^ ^]^ They also put forward the hypothesis that the K^+^ ion likely plays a stabilizing role in the other orthologs as well but has not been found due to differences in the crystallization conditions. Thus, we decided to investigate whether the presence of the full triad, including the K^+^ ion, makes a difference to our simulations of 5U3G and the binding of Gd^+^ to the pocket. The similarity in the binding region made it quite straightforward to transfer the ion triad arrangement from 7MLW and implant it into 5U3G. The positioning of the ion triad is shown in Figure 1, with K^+^ in violet and Mg^2+^ at the binding pocket in green next to the Gd^+^ ligand in blue.

In the following, we show how the transfer of this specific ion configuration significantly stabilizes the ligand binding to the Gd‐I RS in our simulations. The MD simulations provide an atomistic, dynamic picture of the role that the K^+^ from the metal triad plays in the conformational states of the binding pocket via specific, localized interactions – in support of the hypotheses of Trachman et al.^[^ ^8^ ^]^ Moreover, we show that seemingly minor changes in the overall ion setup, such as the positioning of the ions and choice of ion species in solution, may have (possibly unexpected) effects on the conclusions drawn from simulations of highly flexible and highly charged macromolecules such as RNA.

## 2 Results and Discussion

### 2.1 Simulation Starting Structures

In order to investigate the ligand binding dynamics of the Gd‐I RS from *D. dadantii*, we set up the RNA based on the crystal structure 5U3G published by Battaglia et al. in 2017.^[^ ^6^ ^]^ The starting structure for the simulation is shown in Figure 1B, including the bound Gd^+^ ligand (shown in blue) and 24 Mg^2+^ ions (shown in green) that are present in 5U3G. We subsequently solvated the system and added Mg^2+^, Na^+^, and Cl^−^ ions corresponding to the crystallization conditions, see **Table** **1**. After careful equilibration (see Computational Details), we simulated four replicates for 2 µs each. As shown in detail below, this simulation setup led to unstable ligand binding. Following the observation made by Trachman et al.,^[^ ^8^ ^]^ that a K^+^ ion forming a triad with two Mg^2+^‐ions is central to stabilizing ligand binding in an orthologous Gd‐I RS (7MLW), we decided to investigate whether this triad has a stabilizing effect on the 5U3G structure as well. Because the binding pockets of the two orthologs are highly conserved, it was possible to transfer the ion configuration from 7MLW to the pocket of 5U3G. The resulting starting structure is shown in Figure 1C. While the RNA (tan) and the ligand position (blue) were left unchanged, the bound (sometimes also denoted as chelated) ions from the 7MLW structure were transferred to 5U3G. In the immediate vicinity of the Gd^+^ ligand, that transfer entailed the addition of the K^+^ ion and a minor repositioning of two Mg^2+^ ions. Details of the binding pocket of 5U3G with the ion triad are shown in Figure 1D. The pocket has four sides where the RNA comes in contact with Gd^+^: The pocket floor is formed by nucleobase G63, which interacts with the planar face of Gd^+^ via a cation‐π stacking. The Hoogsteen edge of G81 forms two hydrogen bonds with one edge of Gd^+^, while two oxygen atoms (a non‐bridging phosphate oxygen and the O5' from G64) form hydrogen bonds with its second edge. The third edge of Gd^+^ is bound via dipole–dipole interactions with the residues A34 (via N1) and G33 (via O6), which are referred to as the AG‐bulge (indicated as red sticks in Figure 1A–C). The AG‐bulge residues serve an additional function in that they flip out of the otherwise continuously stacked, so‐called P1 loop (a double helix constituting the left side of the RS structure, indicated by the left dashed box in Figure 1A) and protrude into the so‐called P3 loop (constituting the right side of the RS structure indicated by the right dashed box in Figure 1A) to form part of the pocket. In this way, they connect P1 to P3, which is the likely start of the signaling cascade in this RS. As shown in the figure, the triad ions sit above the AG‐bulge, with K^+^ coordinated by A34. Note that the residue numbering used here for 5U3G corresponds to the numbering in the deposited crystal structure and deviates from the publication^[^ ^6^ ^]^ by four residues. The RNA structure of 5U3G with the transferred ions from 7MLW was solvated and K^+^ and Cl^−^ ions were added corresponding to the crystallization conditions, see Table 1. After applying an equilibration protocol analogous to the 5U3G simulations with unaltered ionic setup, we simulated six replicates of this modified system for 2 µs each.

**Table 1:** Simulated systems. Starting PDB structure, presence, or absence of Gd^+^ ligand, ion composition including bound ions, diffuse ions, and solvent.

Starting PDB	Ligand	Triad K^+^	Bound ions	Diffuse ions	Water	Simulation Time
5U3G	w/ Gd^+^	no K^+^	5U3G ‐ 24 Mg^2+^/1 Na^+^	43 Mg^2+^/44 Na^+^/96 Cl^−^	47045	4 × 2 µs
5U3G	w/ Gd^+^	K^+^ placed	7MLW ‐ 4 Mg^2+^/3 K^+^	155 K^+^/83 Cl^−^	44512	6 × 2 µs
5U3G	w/o Gd^+^	no K^+^	5U3G ‐ 24 Mg^2+^/1 Na^+^	44 Mg^2+^/44 Na^+^/97 Cl^−^	47045	4 × 2 µs
5U3G	w/o Gd^+^	K^+^ placed	7MLW ‐ 4 Mg^2+^/3 K^+^	156 K^+^/ 83 Cl^−^	44512	3 × 2 µs

### 2.2 Transfer of Ion Configuration Stabilizes Ligand Binding by Stabilizing the Binding Pocket

**Figure 2:**
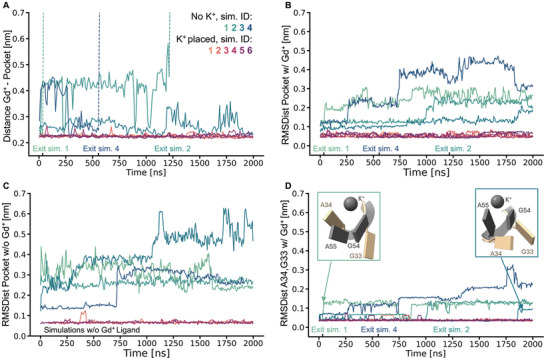
A) Distance of Gd^+^ ligand to binding pocket over time for all simulation replicates. Simulations with original ion setup from 5U3G are colored in tones of green and blue, simulations with the transferred ion setup are red and purple. The time points of Gd^+^ exiting the pocket (distance > 0.6 nm) are marked at the time axis for the corresponding simulations. B) Root mean square deviation of pairwise distances (RMSDist) within the binding pocket over time, relative to starting structure, coloring as above. C) RMSDist of the pocket for simulations in which Gd^+^ was removed prior to simulating, relative to starting structure, ion setups were otherwise equivalent, coloring as above. D) RMSDist of the AG‐bulge (residues A34, G33), relative to 7MLW, for simulations with Gd^+^, coloring as above. Insets show select timesteps from simulation 1 (green) and 3 (blue) to illustrate the variability of the AG‐bulge. Simulated residues are shown in tan, 7MLW is shown in gray for reference. For clarity, all time series are displayed as running averages over 100 frames (10 ns) for panel A and 50 frames (5 ns) for panels C‐D.

**Figure** **2**A shows the distance of the ligand from the center of the binding pocket for all simulations. The four simulations with the original ion setup from 5U3G (i.e., the ones missing the K^+^ ion of the triad) are colored in blue and green hues, the six simulations with the transferred ion setup (i.e., with the complete triad including K^+^) are colored in red and purple hues. As already mentioned, the simulations with the original ion setup from 5U3G did not display stable ligand binding. In three of the four simulations, the Gd^+^ ligand left the binding pocket fully. In those cases, no rebinding to the Gd^+^ binding pocket was observed in the course of our simulations. The respective exits are marked at the time points where the ligand‐pocket distance exceeded a value of 0.6 nm (from there on the distance trace are hidden for clarity). In the fourth simulation, the ligand did not fully leave the RNA binding pocket, yet it displayed substantial mobility. In summary, the ligand does not seem to find a stable bound position in the simulations of 5U3G, in spite of applying a very cautious equilibration protocol to avoid disturbances due to the initial structure. In contrast, the ligand stayed fully bound for the entirety of the simulation in each of the replicas with the transferred ion setup. This can be seen in Figure 2A: The Gd^+^‐pocket distance fluctuates very stably around a value of 0.23 nm for all simulations in which the triad was transferred (red and purple hues). Thus, not only does the ligand stay bound, it also appears to be much more immobilized inside the pocket compared to the one simulation without the triad K^+^ where Gd^+^ does not leave (no K^+^, sim ID 3). While we had hoped for the addition of the triad K^+^ to have a stabilizing effect, we were nevertheless surprised by its extent. To better understand this result, we investigated the effect of transferring the ion configuration on the binding pocket, specifically its flexibility. To this end, the root mean square deviation of all pairwise distances (RMSDist) between RNA atoms that are involved in ligand binding (see Computational Details) was computed over the duration of each simulation (see Figure 2B). Comparing the simulations with the original ion setup from 5U3G (no triad K^+^) to those with the transferred ion setup shows clearly that the ion transfer strongly reduces the relative flexibility of the pocket atoms, effectively making the pocket more stable. While the RMSDist values increase for all simulations with the original ion configuration, they stay consistently low for all simulations in which the ion triad was transferred. For comparison, we also performed this analysis for simulations in which the Gd^+^ ligand had been removed initially (see Figure 2C). Here, a similar effect is observed, indicating that the stabilizing influence of the full ion triad, i.e., the presence of the K^+^, is an effect on the binding pocket itself, not tied to the simultaneous presence of the ligand. In summary, this can be interpreted as the ion triad being necessary and sufficient for stabilizing the binding pocket.

### 2.3 K^+^ Continuously Occupies the Triad Position

**Figure 3:**
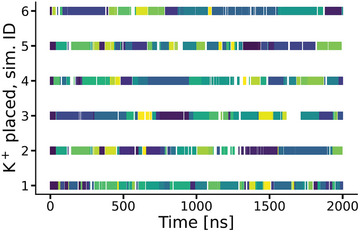
Existence plot of a K^+^ ion in proximity (within a cutoff distance of 0.6 Å) to both A34 and G33. Each row shows one of the six simulations of 5U3G with the triad K^+^ placed. Colors have been arbitrarily assigned to the IDs of the (in total 158) K^+^ ions so that a change of color indicates an exchange of K^+^. White space indicates that no K^+^ was found within cutoff distance.

In a next step, the binding of the K^+^ ion in the triad position is analyzed. **Figure** **3** shows that the K^+^ position in the triad is consistently filled with an ion in all six simulations where a K^+^ had been initially positioned in the triad site (i.e., with the transferred ion setup). The data also show that this K^+^ ion fairly rapidly and frequently exchanges with free K^+^ ions from the solution. While there are stretches in the trajectories where the site is empty, they are fairly short. In order to facilitate this exchange and the rapid refilling of the triad site, a relatively high K^+^‐concentration in solution is required. To validate that this is relevant, we performed additional simulations where we transferred only the triad (i.e., put a single K^+^ ion at the triad position) but did not add free K^+^ ions to the solution (instead keeping the high Mg^2+^‐concentration of the 5U3G crystallization conditions). In those simulations, the K^+^ quickly left its position and did not rebind. Afterward the system behaved like in the 5U3G simulations without transfer of ion setup, i.e., with all the consequences on the stability of the binding pocket and the binding of the Gd^+^ ligand (data not shown). This observation is highly relevant for two reasons. While the K^+^ in the triad position obviously has a critical stabilizing effect on the structure, it is by no means statically bound, but very dynamic. It also shows that the mere transfer of the ion triad alone would not have sufficed to observe stable Gd^+^ binding for a sustained amount of time, i.e., the conclusions from the simulations would have been quite different.

### 2.4 K^+^ Plays a Crucial Role in Stabilizing Ligand Binding

The fact that we have obtained simulations where the Gd^+^ ligand remained stably bound to the RS gave us the opportunity to investigate the binding situation in more detail. By contrasting the simulations including K^+^ (i.e., with the ion triad transferred) to the simulations where K^+^ is missing, we can determine its role in keeping the ligand in the pocket. While we have already described that the entire binding pocket is significantly more flexible when the triad K^+^ is not present, there are two residues in the binding pocket that play a particular role in the functioning of the RS and that warrant a closer look: as described above and illustrated in Figure 1, the residues A34 and G33 are flipped out of the helix of the P1 loop and form an asymmetric dinucleotide bulge (AG‐bulge) that forms part of the binding pocket. The K^+^ of the ion triad sits in close proximity to this AG‐bulge. More specifically, K^+^ is coordinated by residue A34 from the AG‐bulge. In their analysis of the 7MLW crystal structure (with triad), Trachman et al. show that the coordination of K^+^ shifts the position of A34 in relation to the 5U3G structure (without K^+^).^[^ ^8^ ^]^ This puts this base in a position that enables it to form a tighter dipole–dipole interaction with the ligand via N1. The residue G33, which is stacked parallel to A34 also forms dipole–dipole interactions with Gd^+^ via O6. In addition to stabilizing the ligand binding by forming favorable interactions, the AG‐bulge also sterically constricts the ligand binding pocket – a function that not only serves the sterical discrimination against larger Gd^+^‐derivates, but also supports the retention of the ligand in the pocket by tightening the pocket entrance.

With this analysis of the crystal structure in mind, we looked more closely into the structural role of the triad K^+^ ion in the simulations and the structure and dynamics of the RNA in its vicinity. In particular, we analyze what happens structurally to the binding pocket, when the triad K^+^ ion is missing. While Figure 2B is concerned with the overall structural stability of the binding pocket (via the RMSDist of all pairwise distances of the pocket), Figure 2D more specifically focuses on the structural stability of the AG‐bulge. Here, the RMSDist of the AG‐bulge residues (G33, A34), in reference to the corresponding AG‐bulge in 7MLW (G54, A55). In the simulations where the triad K^+^ ion is absent, the AG‐bulge shows a substantially increased flexibility compared to the ones in which K^+^ is present and coordinated by A34. Importantly, the RMSDist time series in the no‐K^+^ simulations exhibit jumps after which the RMSDist does not revert to the original levels (which corresponds to the intact pocket configuration as observed in the simulations with K^+^). This indicates that there are – on the timescale of the simulations irreversible – conformational changes, which occur in the AG‐bulge. The trajectories differ with respect to the timing of these RMSDist jumps. Simulation 1 (green line) shows a relatively early event, which corresponds to the ligand leaving the binding pocket very early in the simulation. The inset with green outline in Figure 2D shows the extent to which the AG‐bulge has changed conformation already at 4.7 ns. The AG‐residues from the simulation are shown in tan, the corresponding residues from the reference 7MLW and the K^+^ at the triad site are shown in gray. While A34 has moved up and into a position it would not be able to access if K^+^ were present, G33 has moved down, losing its interactions with Gd^+^. Simulation 3 (the simulation where the Gd^+^ remains in the pocket, albeit rather dynamically) shows a different scenario. Here, the AG‐bulge remains fairly rigid for most of the duration of the simulation, whereas the pocket displays some flexibility (see Figure 2B). In the last 200 ns the AG‐bulge RMSDist shows a jump. The extent of the motions of the AG‐bulge is shown in the inset with the blue outline. Both A34 and G33 have dropped down and out of their functional position (compared to 7MLW shown in gray).

**Figure 4:**
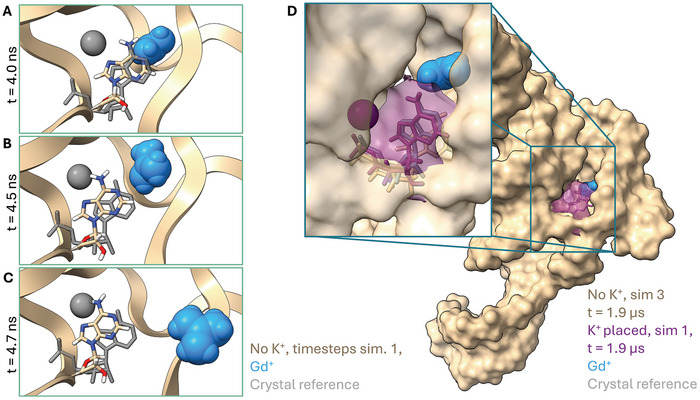
A – C) Three frames of Gd^+^ (blue) leaving the binding pocket in simulation 1 with original ion setup (no K^+^). D) Timestep (1.9 µs) of simulation 3 with original ion setup (no K^+^, (tan)) overlaid with timestep (1.9 µs) of simulation 1 with K^+^ placed in triad position (violet, only A34 and K^+^ shown) For reference, the 7MLW crystal structure is shown in gray.

The early unbinding event in simulation 1 is shown in more detail in **Figure** **4**A–C: At 4.0 ns (Figure 4A), the ligand is embedded in the binding pocket. A34 is in a position similar to A55 in 7MLW (gray reference), but there is no K^+^ to keep it in place. At 4.5 ns (Figure 4B), A34 has moved upward, opening the binding pocket, allowing Gd^+^ to unbind. Figure 4C shows the ligand leaving the pocket at 4.7 ns. By comparing the position of A34 at the timesteps 4.5 ns and 4.7 ns to the position of A55 and K^+^ in 7MLW, it becomes evident that this opening of the pocket would have introduced steric clashes between K^+^ and A34, if K^+^ had been present at the triad site.

Figure 4D shows a further illustration of the instability of the AG‐bulge when the triad‐K^+^ is missing. Here, a single timestep (t = 1.9 µs) of simulation 3 without K^+^ is shown in tan. It can be seen from the inset, that A34 (tan wire representation) has dropped down and no longer interacts with the ligand (blue). To contrast this, we show the position of A34 (violet) at an equivalent timestep from a simulation where the triad K^+^ (violet) had been placed. While A34 in the simulation without K^+^ moved from its position and opened up the pocket substantially, it stays in place in the simulation with K^+^. A comparison with A55 from 7MLW (gray wire representation) shows that in the presence of K^+^, A34 adopts a position where it can interact with the Gd^+^ ligand. In summary, in presence of the triad K^+^ the A34 and G33 residues of the AG‐bulge remain in a position where they can form and retain stabilizing contacts with the Gd^+^ ligand and can fulfil the role of constraining the pocket and retaining the ligand.

### 2.5 Exchange of Ion Setup Increases Global Flexibility While Locally Stabilizing the Binding Pocket

**Figure 5:**
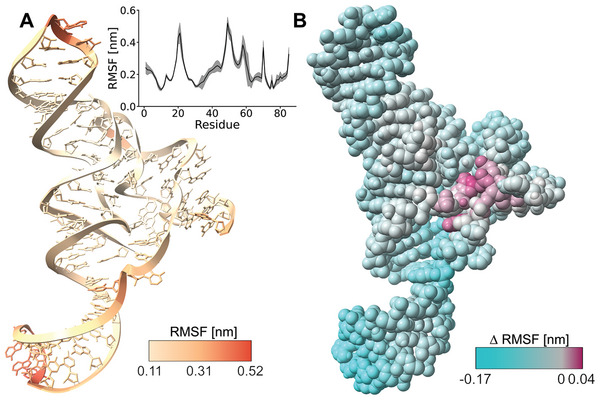
A) Average root mean square fluctuation (RMSF) of all simulations with transferred ion setup (K^+^ placed, high K^+^‐concentration), calculated per residue over the entire duration of the simulations. Inset: Graph of average RMSF, shaded area represents the standard deviation between simulations. B) Difference in average RMSF (ΔRMSF) between all simulations with original ion setup (no K^+^, high Mg^2+^‐concentration) and transferred ion setup (K^+^ placed, high K^+^‐concentration), calculated per atom, with a windowed average with 50 ns window‐size. Negative values (cyan) indicate an increased flexibility upon changing the ion setup, positive values (purple) indicate a decreased flexibility upon changing the ion setup. Gray indicates little to no change.

We have seen that the localized placement of an individual ion at a functionally distinguished site can have a substantial impact on an RNA system to the extent of stabilizing a bound ligand, which had so far appeared “curiously” poorly bound in the simulations. However, it should be noted that the transfer of the ion setup from 7MLW to 5U3G entailed not only the placement of a K^+^ ion at the triad site. It also included a change in the ion setup in the solution due to different crystallization conditions. As already pointed out, the exchange of the predominant solvent ions from Mg^2+^ to K^+^ had an important impact on whether enough K^+^ was available to keep the triad position filled. The different crystallization conditions had an effect on the ions that were co‐crystallized with the RNA in the two crystal structures. In consequence, the transfer of the ion setup also included the removal of most of the Mg^2+^ ions bound to the RNA scaffold in 5U3G, which becomes evident in comparing the two starting structures (Figure 1B,C). However, the removal of bound Mg^2+^ ions is expected to bring with it a loss of structural rigidity in the vicinity of the Mg^2+^ ion binding sites due to a loss of ion‐bridges formed by this divalent ion.^[^ ^23^, ^24^, ^25^ ^]^ To investigate this, we calculated the root mean square fluctuation (RMSF) of the RS. **Figure** **5**A shows absolute RMSF values of all simulations with the transferred ion setup, averaged over the full length of the simulations. This gives a perspective of the absolute flexibility and of larger motions of different regions of the RS at longer timescales. It shows that in spite of removing the bound Mg^2+^ ions the structural integrity of the RS is maintained over the entire duration of the simulation, with the most flexible regions being the solvent exposed residues at the top and bottom of the RS. However, these relatively large motions of residues that do not contribute directly to the switching of the RS obscure the impact of the change of ion conditions on the binding pocket. Here, the differences are more subtle and manifest in changes in residue fluctuations on shorter timescales. To visualize this, we calculated the RMSF difference between the simulations with the original ion setup and those with the transferred ion setup based on a windowed RMSF calculation with a window‐size of 50 ns (Figure 5B). Negative (cyan) values mean that the overall flexibility in the region of a residue has increased upon changing the ionic setup, while positive (purple) values indicate that the flexibility is reduced. It is evident that some regions of the RS gain flexibility upon being stripped of their Mg^2+^ ions, while other regions are not strongly affected. It is however notable that despite a general trend of increased flexibility in the RNA overall, there is a localized rigidification at the binding pocket, which is where the triad K^+^ was placed. This underscores our previous conclusion that the specific positioning of ions can have unexpectedly strong effects on the dynamics of charged biomolecules – and makes the effect around the binding pocket all the more remarkable.

## 3 Conclusion

Here, we have studied the effects of the ionic makeup of a large and complex (functional, folded) polyelectrolyte system on its stability, structural integrity, and function for the case of the Gd‐I RS. We have shown that the careful placement of an individual ion can have an all‐or‐nothing effect on the entire macromolecular system, affecting whether the atomistic simulations generally agree with the experimental observation that this RS stably binds the Gd^+^ ligand. Specifically, transferring the ion setup from the crystal structure of another Gd‐I RS ortholog stabilized the ligand binding in the 5U3G system to an extent that made it possible to study the dynamics of the RS in more detail. This enabled us to discern the specific role that K^+^ played in the metal ion triad that was transferred. Here, the simulations confirm observations that had been previously stated based on a careful comparison of the different Gd‐I RS crystal structures,^[^ ^8^ ^]^ namely that the full cation triad including the K^+^ ion has an important role in stabilizing the binding pocket. This affects both the binding of Gd^+^ and the conformations of the AG‐bulge residues which in turn are relevant for the overall 3‐dimensional fold of the switched conformation.

The MD simulations have proven to be a highly valuable tool to support such structural analyses, providing a more nuanced picture of contributing interactions and the influence of individual factors on fluctuations, conformational equilibria and the dynamics of transitions. There are however, other potentially impactful factors influencing the structure and function of the Gd‐I RS. For example, investigating the binding behavior of other ligands, such as uncharged Gd^+^ analogs like urea, which has shown substantial differences in simulations of the Gd‐II RS,^[^ ^22^ ^]^ allows to dissect the influence of factors such as ligand charge or shape. Extending the study to other, more recent force fields that include further parameter optimizations^[^ ^15^, ^26^, ^27^, ^28^ ^]^ can yield additional insights into the nuanced interactions in this structural RNA and can corroborate or complement the findings reported here. After all, our results demonstrate that for biomacromolecules – in particular in the case of polyelectrolytes such as RNA or DNA – simulating potentially very specific, localized conformational effects associated with folding or binding is highly sensitive to details regarding the simulation setup. These include global factors such as the concentrations of different ionic species in solution down to very local ones such as the presence/absence of individual ions at critical positions, which are not necessarily obvious a priori.^[^ ^29^ ^]^ In particular, we note the impact of the availability of diffuse K^+^ ions on whether stable Gd^+^ binding is observed at all in the simulations. This nicely ties to other observations^[^ ^24^, ^25^, ^30^, ^31^ ^]^ that the choice of cation species in the investigation – both computational and experimental – of such biological polyelectrolytes should not be underestimated.

## 4 Computational Details

### Molecular Dynamics Simulations

The Guanidine‐I RS aptamer structure *Dda_ykkC* from *Dickeya dadantii* (PDB‐ID: 5U3G)^[^ ^6^ ^]^ was simulated with various ion setups and in presence and absence of the guanidinium ligand (Gd^+^). As an additional source for the ion configuration, the orthologous Gd‐I aptamer from *Burkholderia sp. TJI49* (PDB‐Id: 7MLW)^[^ ^8^ ^]^ was used. Whereas 5U3G includes bound Mg^2+^ ions and a Na^+^ ion, 7MLW included – besides Mg^2+^ ions – additional bound K^+^ ions. Additionally, both differ in the crystallization conditions: A high Mg^2+^ concentration in the case of 5U3G and a high K^+^ concentration in the case of 7MLW. Initially, simulations of the 5U3G structures were performed under high magnesium conditions, which displayed unstable Gd^+^ binding. As the ion triad was reported to stabilize the aptamer complex, the ion configuration of the 7MLW structure was transplanted into the 5U3G structure by superposition of the structures with ChimeraX.^[^ ^32^ ^]^ One potassium of 7MLW was located outside the overlapping regions between both structure and was therefore omitted. The Sr^2 +^ ions in 7MLW, which were commonly used in RNA crystallization, had been replaced with Mg^2+^ ions, which were also divalent and more abundant under physiological conditions. The triphosphate group at the 3' terminus was removed and the terminus was treated as neutral. The different ion and ligand condition are detailed in Table 1.

All MD simulations were performed using the GROMACS program package version 2022.6.^[^ ^33^ ^]^ Building on a previous study of the Gd‐II riboswitch,^[^ ^22^ ^]^ the AMBER based DESRES nucleic acid force field published by the D. E. Shaw research group^[^ ^34^ ^]^ was applied, along with the TIP4PD water model^[^ ^35^ ^]^ in the implementation provided by Giovanni Bussi, Stefano Piana, and Sandro Bottaro at https://github.com/srnas/ff/tree/desres. The parameters for the Gd^+^ ligand were assigned according to Wernesson et al.^[^ ^36^ ^]^ as previously used for the Gd‐II RS.^[^ ^22^ ^]^ The solute (i.e., RNA structure with and without Gd^+^ ligand and the bound ions) was placed in the middle of a dodecahedron box with a minimum distance of 1.9 nm between RNA and the box wall. The box was solvated and ions were added according to the general composition described in the results section, resulting in the systems reported in Table 1. The following general simulation settings had been applied: electrostatic interactions were calculated with the particle mesh Ewald method (PME),^[^ ^37^, ^38^ ^]^ with a real space and van der Waals cut‐off distance of both 1 nm. For production simulations an integration timestep of 2 fs was used utilizing the leap‐frog integrator. The velocity‐rescale algorithm^[^ ^39^ ^]^ was used for temperature coupling and the Parrinello‐Rahman algorithm^[^ ^40^ ^]^ with a damping constant of 2.0 ps for pressure coupling. Initially the system was energy minimized with the steepest descent algorithm and position restraints of 1000 kJ ${\text{mol}}^{-1}$ ${\text{nm}}^{-2}$ on the solute (RNA, ligand and structural ions). Afterward a sequence of equilibration simulations was performed: 50 ps at NVT conditions with a reduced timestep of 1 fs and position restraints of 1000 kJ ${\text{mol}}^{-1}$ ${\text{nm}}^{-2}$ applied to the solute, followed by a series of 50 ps long simulations at NPT conditions with decreasing strength of position restraints (1000, 750, 500, 250, 0 kJ ${\text{mol}}^{-1}$ ${\text{nm}}^{-2}$). Finally, 2 µs long production simulation were performed with different numbers of replicates (see Table 1), where replicates differed in the velocity initialization in the NVT step of the equilibration protocol.

### Analysis

The binding state of the Gd^+^ ligand to the binding pocket was characterized by calculating the minimum distance between the N7 atom of residue G81 and any atom of Gd^+^.

The stability of the binding pocket was described by calculating the root mean square deviation of all pairwise (atom‐atom) distances (RMSDist calculation as implemented in GROMACS) within the binding pocket. In this calculation all atoms of residues 33, 34, 63, 79, 81, and the backbone atoms of residue 64 were included. The differences of those RMSDist values to those of the initial structure of the production simulation were computed. In a similar fashion, the structural changes of the AG‐bulge region were characterized by RMSDist calculation based on the atomic pairwise distances in the residues G33 and A34 (in the case of the 5U3G RS). Here, the reference RMSDist value was computed from the orthologous RS structure 7MLW where the corresponding residues were G54 and A55.

The residence of the K^+^ ion in the triad position was computed by determining the identity of ion(s) that were detected at a minimum distance of 0.6 to atoms N6 and N7 of residue A34 and atom O1P from residue G33.

The root mean square fluctuation (RMSF) of the riboswitch was calculated using the function gmx rmsf from the GROMACS package in two different ways. First, the RMSF was calculated for all simulations with the transferred ion setup (K^+^ placed, lines 2 and 4 in Table 1). The RMSF was calculated per residue and over the full length of each of the nine trajectories. It was then averaged to give the mean RMSF and standard deviation for this simulation set. Second, the difference in RMSF between simulations with differing ion setups (ΔRMSF) was calculated by subtracting the average RMSF in simulations with the transferred ion setup (K^+^ placed, lines 2 and 4 in Table 1) from the average RMSF in simulations with the original ion setup (no K^+^, lines 1 and 3 in Table 1). Here, the RMSF for each trajectory was calculated per atom and with a windowed average using a window size of 50 ns.

## Conflict of Interest

The authors declare no conflict of interest.

## Data Availability

The data that support the findings of this study are available from the corresponding author upon reasonable request.
